# Supplementary material for: Dim green light enables day‐and‐night monitoring of leaf movements
Source: Plant J. 2026 Sep 15;127(5):e71119. doi: 10.1111/tpj.71119 (PMC13578868; doi:10.1111/tpj.71119)

Figure S1. Dim green light does not induce stomatal opening in Arabidopsis or lettuce, with minimal assimilation and no electron transport.

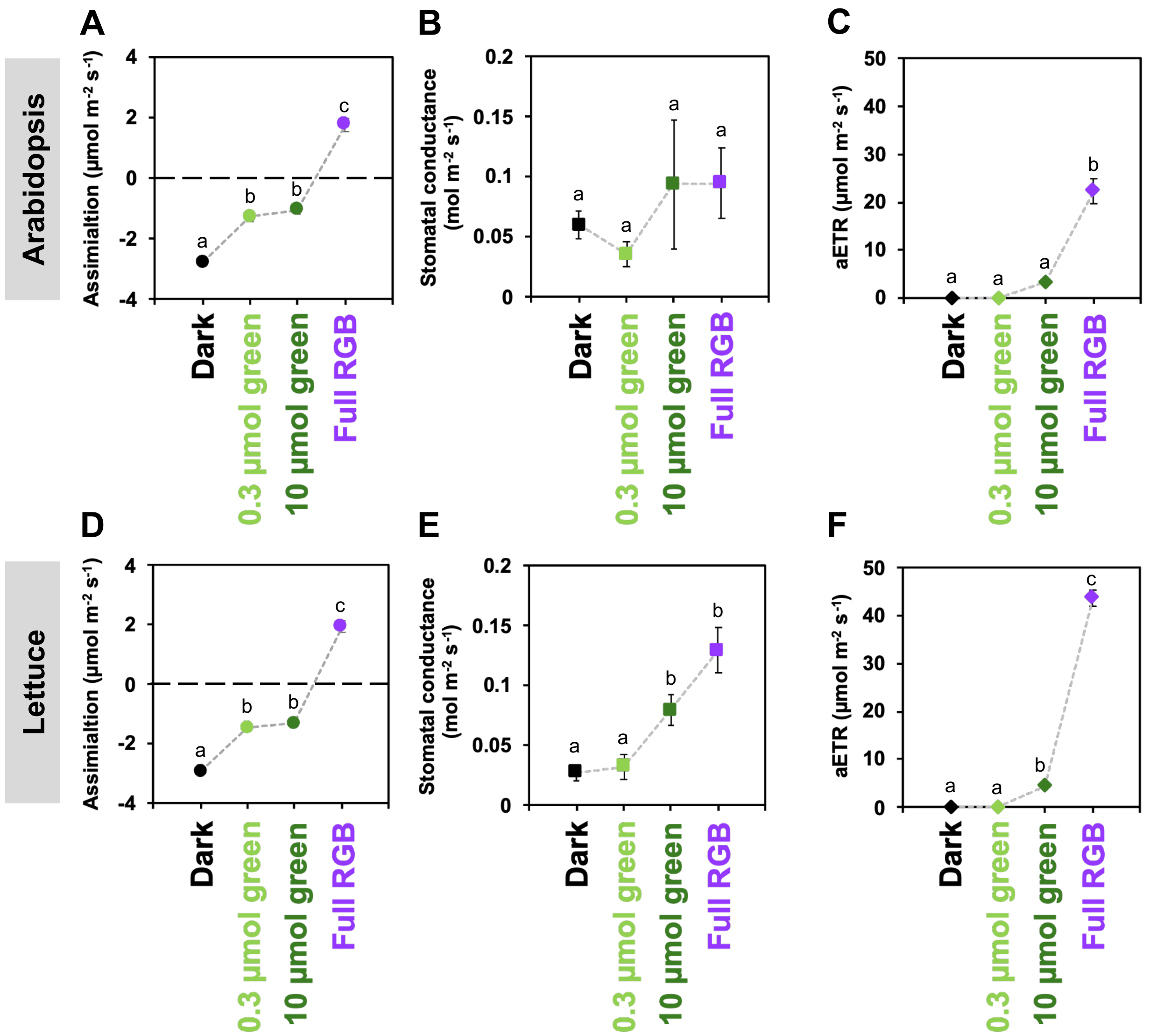

Figure S2. Diel leaf movement in *Arabidopsis* using dimG^{Day_Night} image sequence and TRiP.

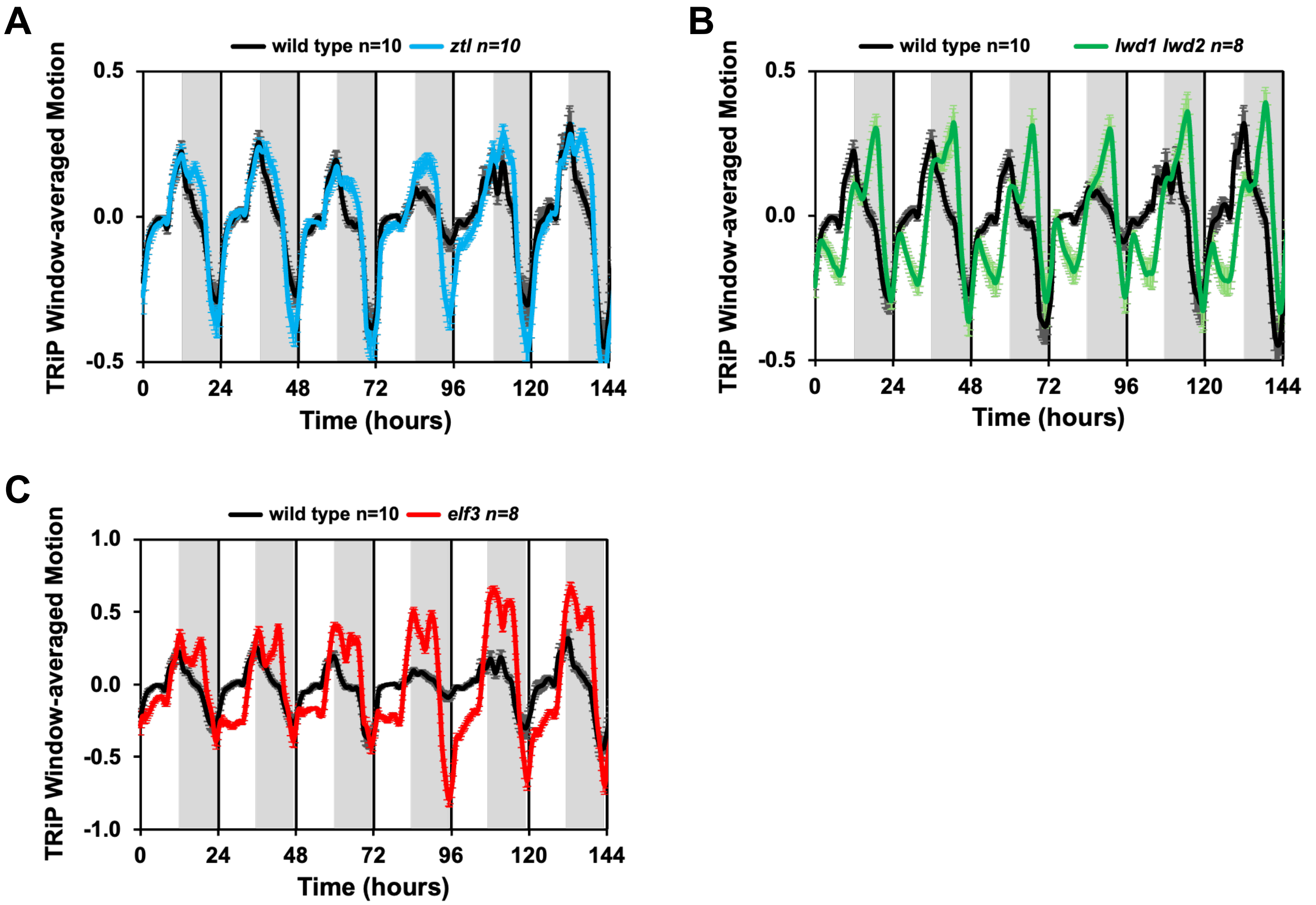

Figure S3 A. Wildtype *w*indow-averaged motion for individual plants using dimG^{Day_Night} and RGB^{Day}_dimG^{Night} image sequences and TRiP.

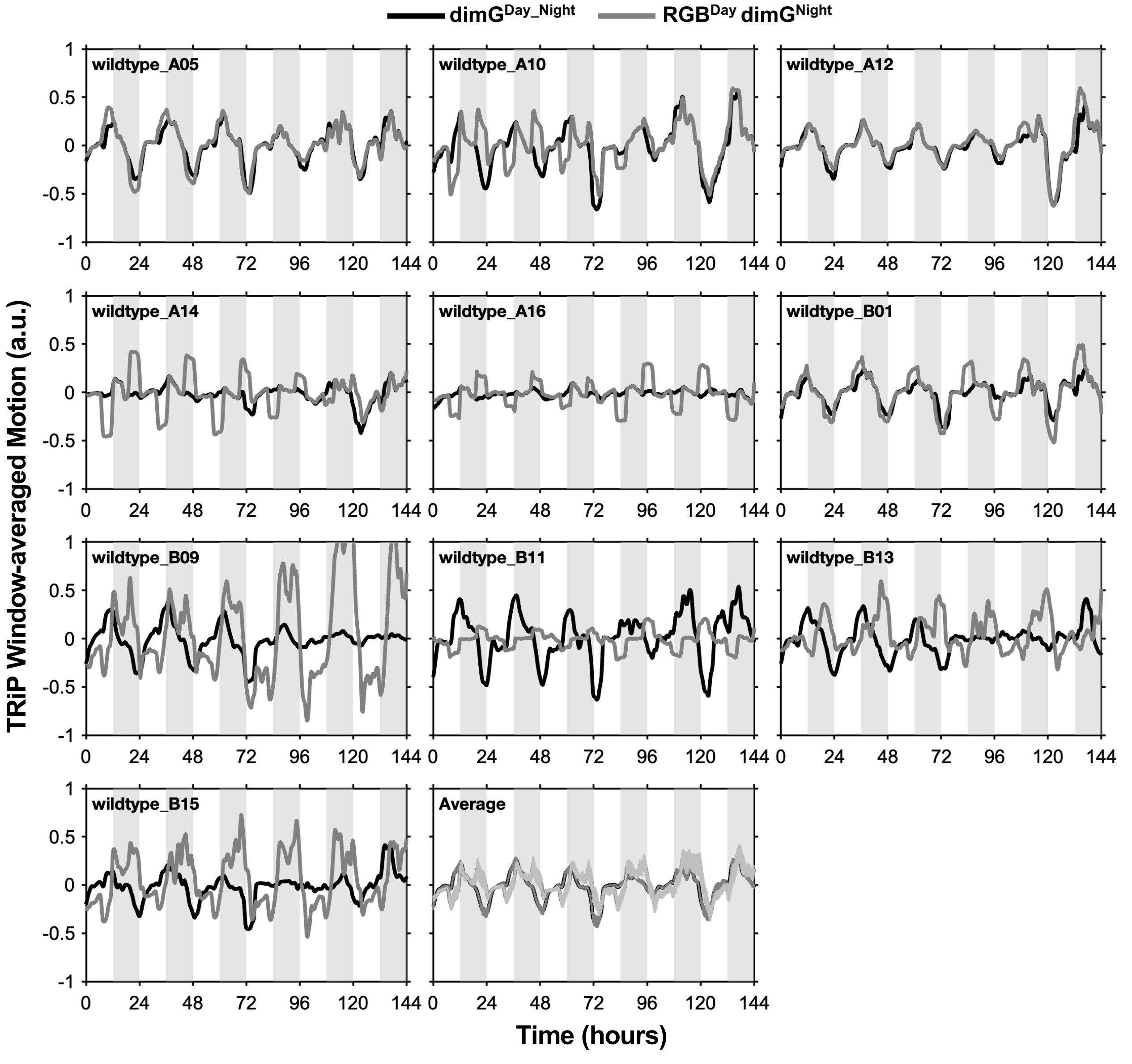

Figure S3 B. *ztl* window-averaged motion for individual plants using dimG^{Day_Night} and RGB^{Day}_dimG^{Night} image sequences and TRiP.

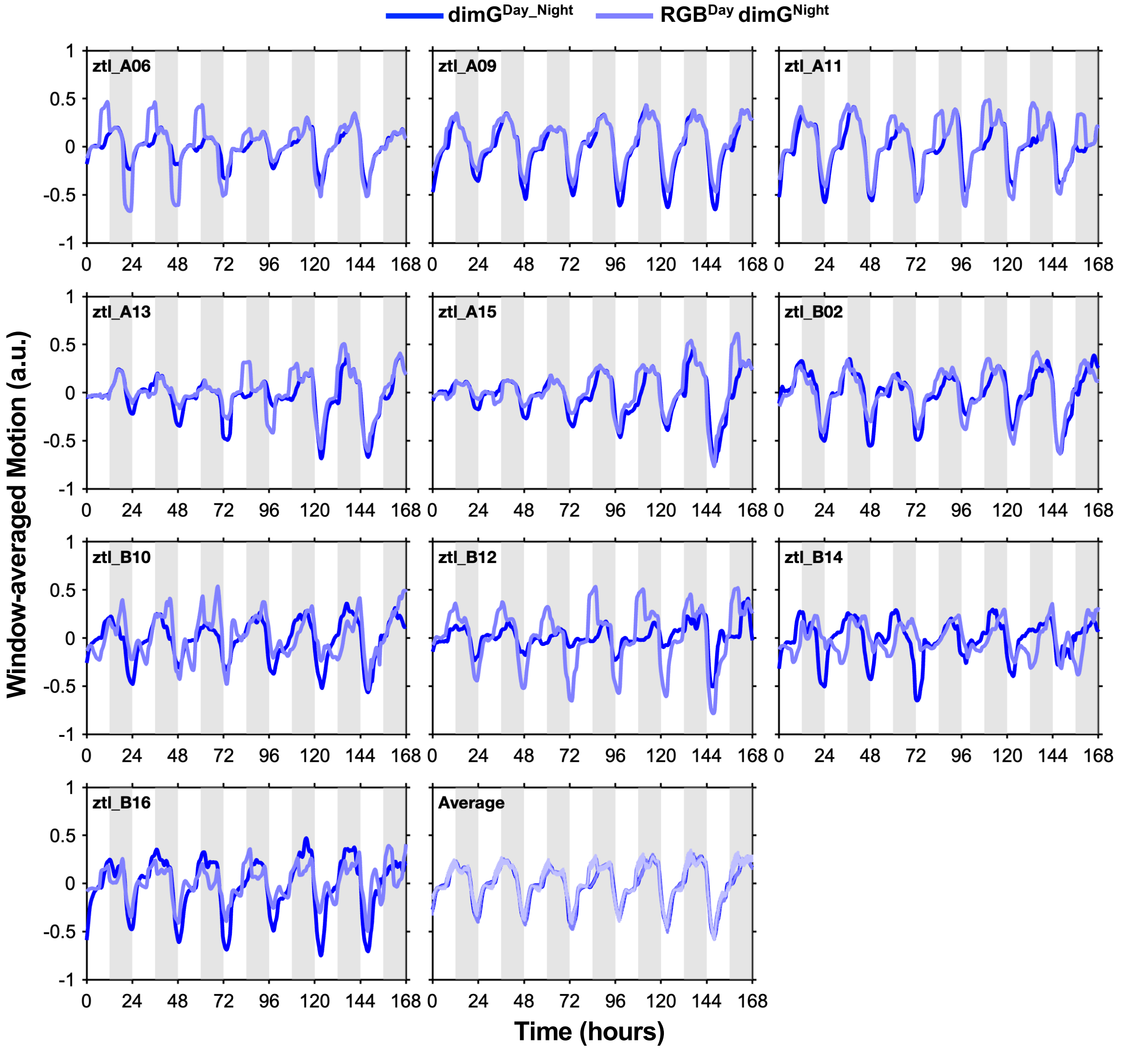

Figure S3 C. *lwd1 lwd2* window-averaged motion for individual plants using $dimG^{Day\_Night}$ and $RGB^{Day}\_dimG^{Night}$ image sequences and TRiP.

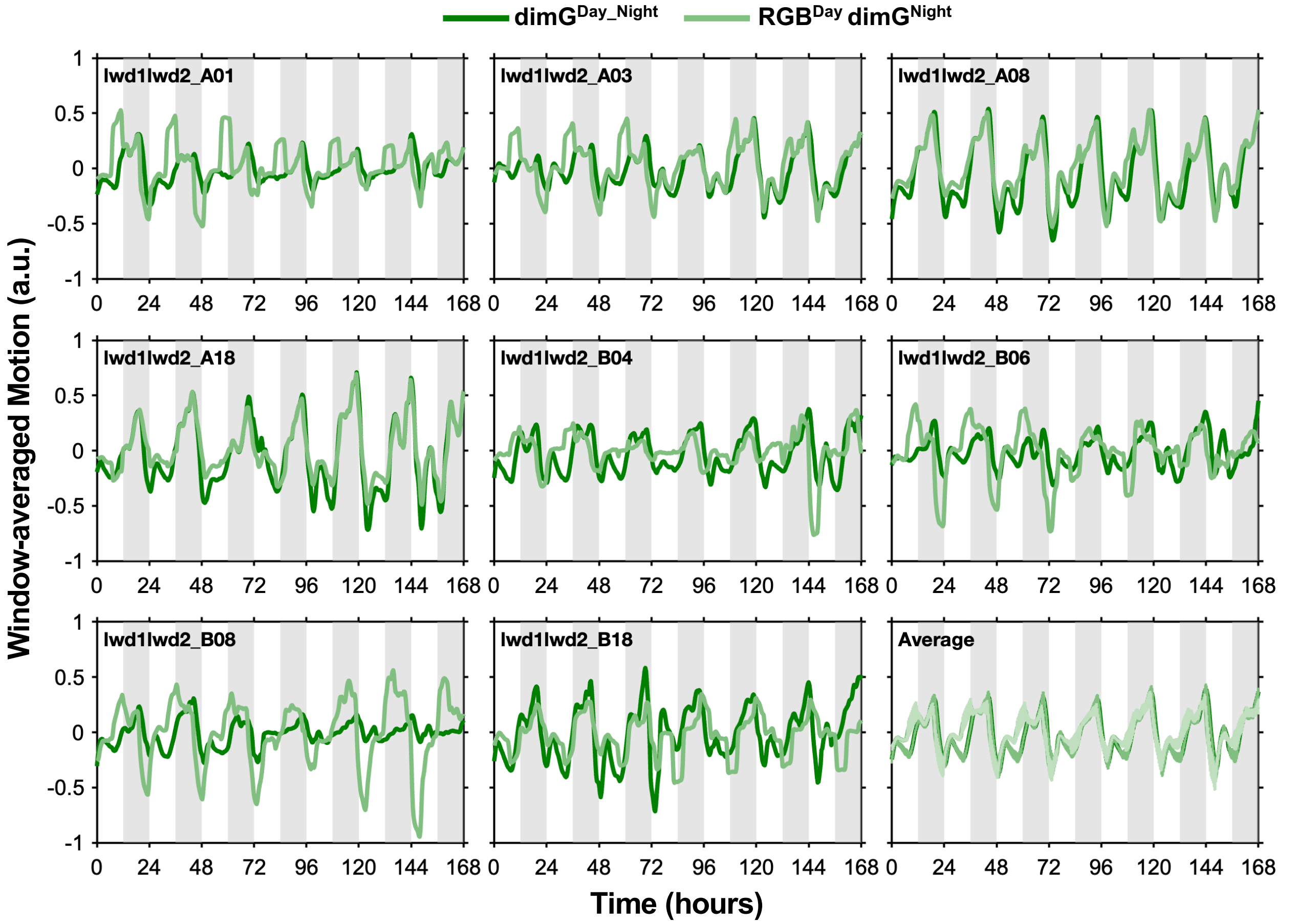

Figure S3 D. elf3-1 window-averaged motion for individual plants using dimG^{Day_Night} and RGB^{Day}_dimG^{Night} image sequences and TRiP.

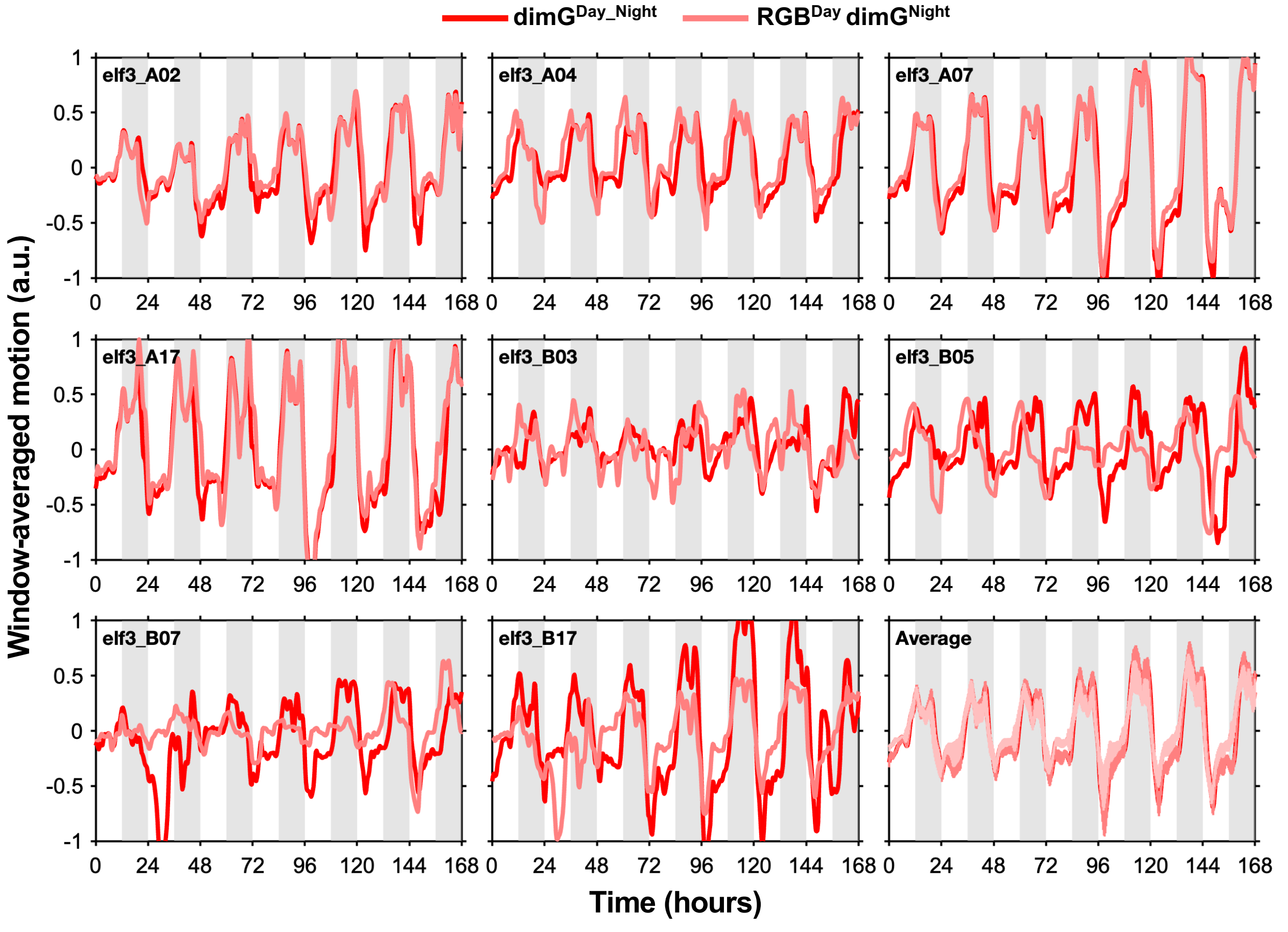

Figure S4 A wildtype diel oscillations of Y-coordinate displacement for individual plants using dimG^{Day_Night} and RGB^{Day}_dimG^{Night} image sequences under ND

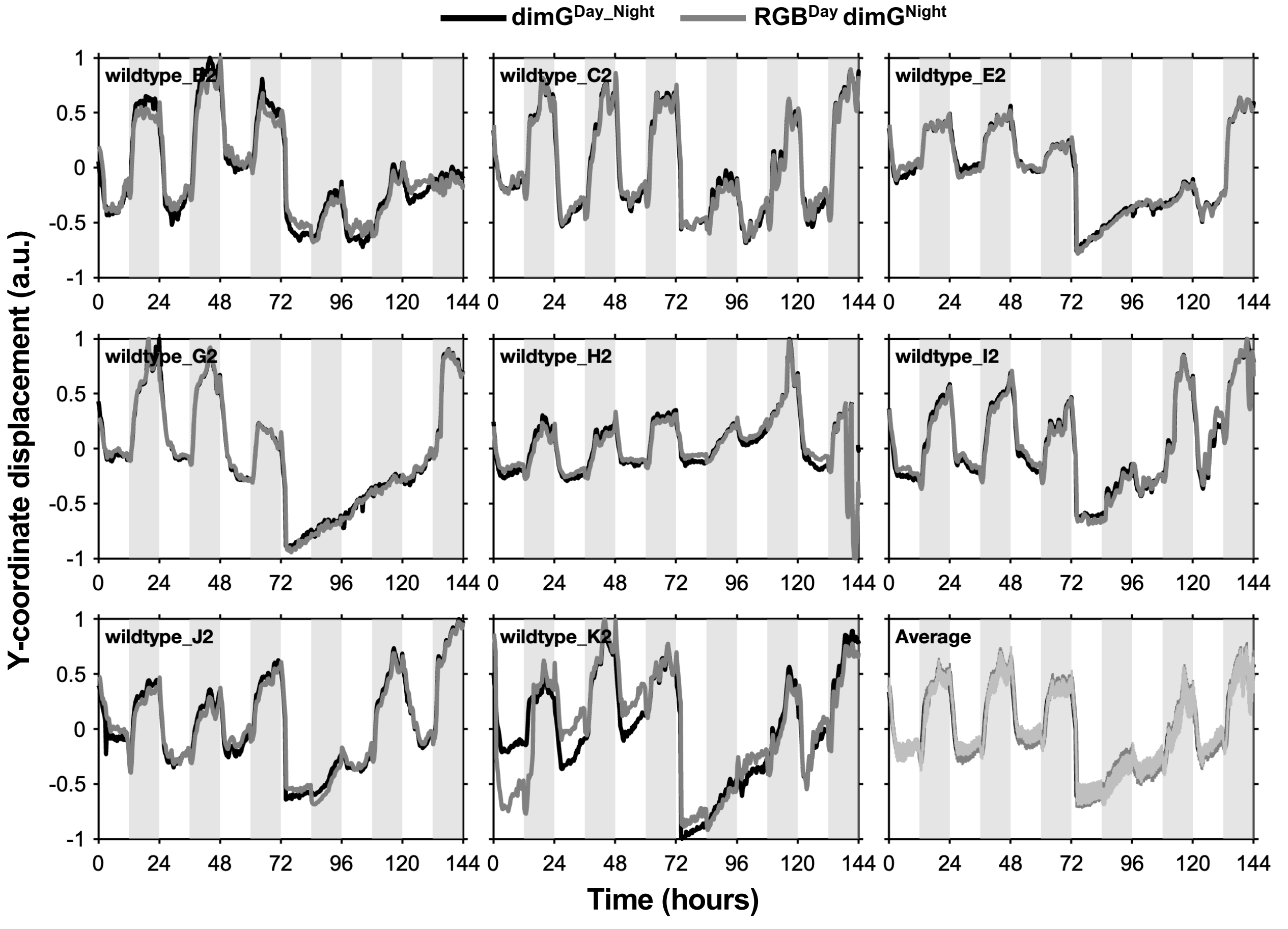

Figure S4 B *ztl* diel oscillations of Y-coordinate displacement for individual plants using $dimG^{Day\_Night}$ and $RGB^{Day}\_dimG^{Night}$ image sequences under ND

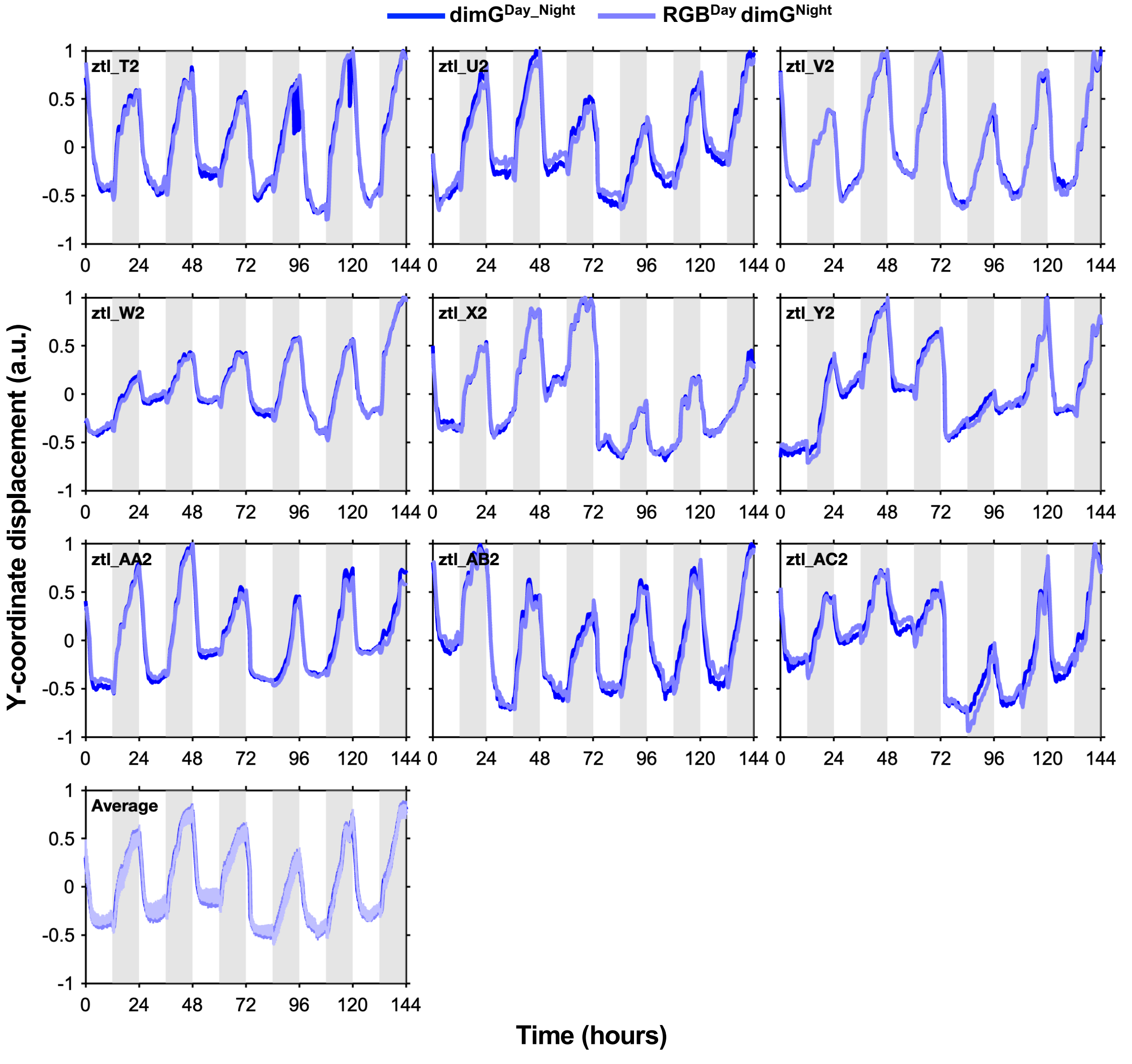

Figure S4 C *lwd1 lwd2* diel oscillations of Y-coordinate displacement for individual plants using dimG^{Day_Night} and RGB^{Day}_dimG^{Night} image sequences under ND

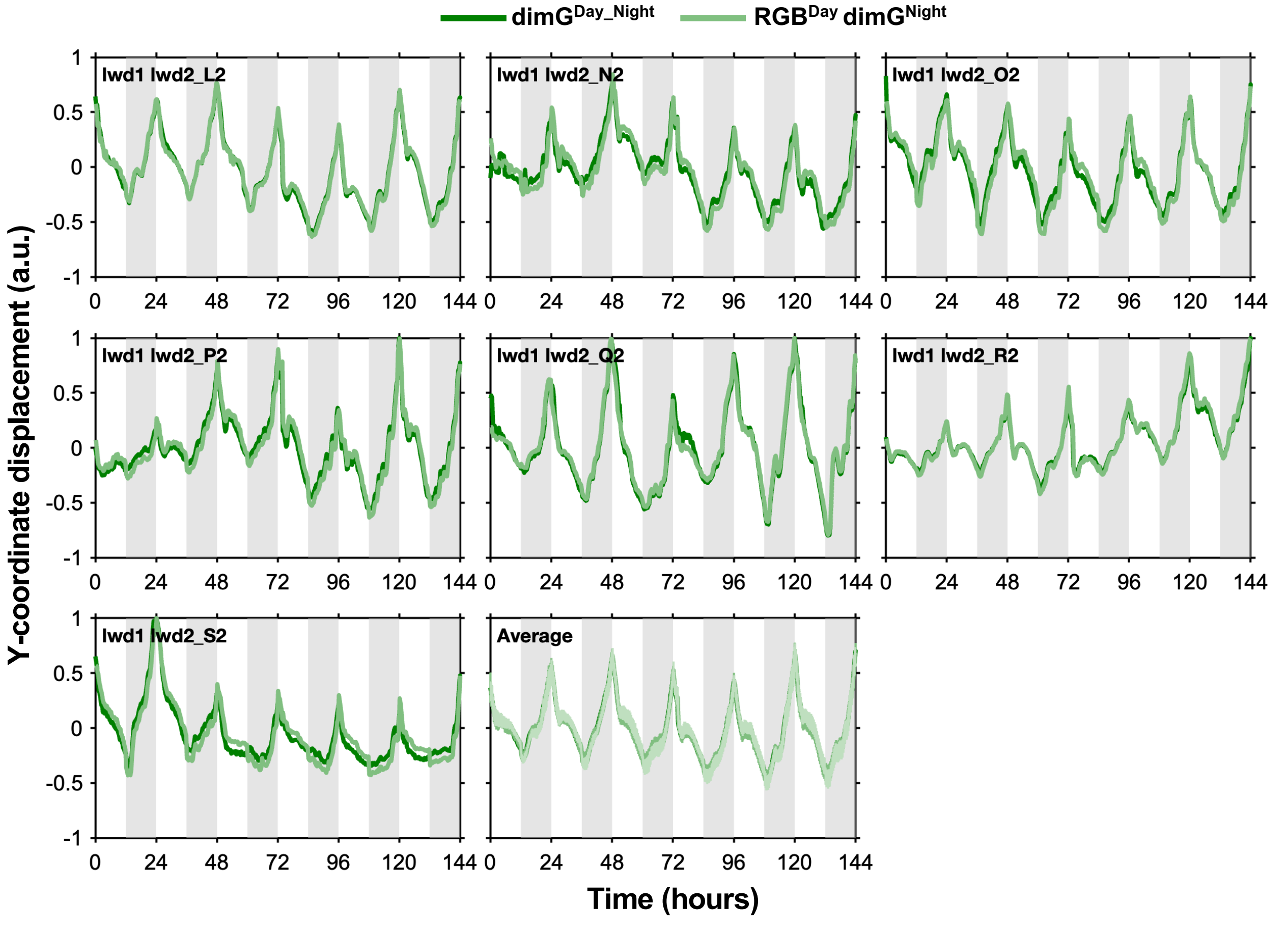

Figure S4 D *elf3* diel oscillations of Y-coordinate displacement for individual plants using dimG^{Day_Night} and RGB^{Day}_dimG^{Night} image sequences under ND

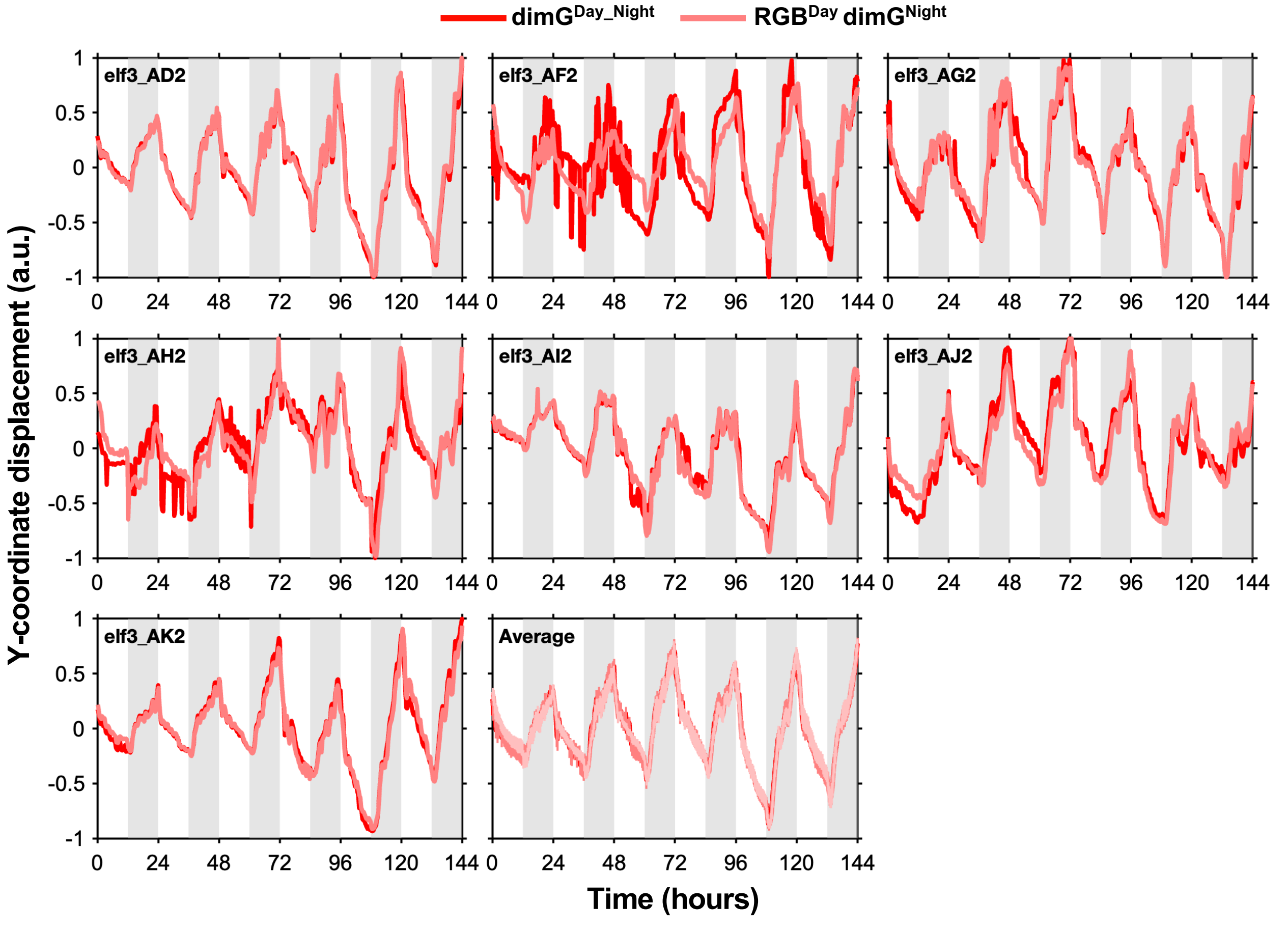

Figure S5 A wildtype diel oscillations of Y-coordinate displacement for individual plants using dimG^{Day_Night} and RGB^{Day}_dimG^{Night} image sequences under LD

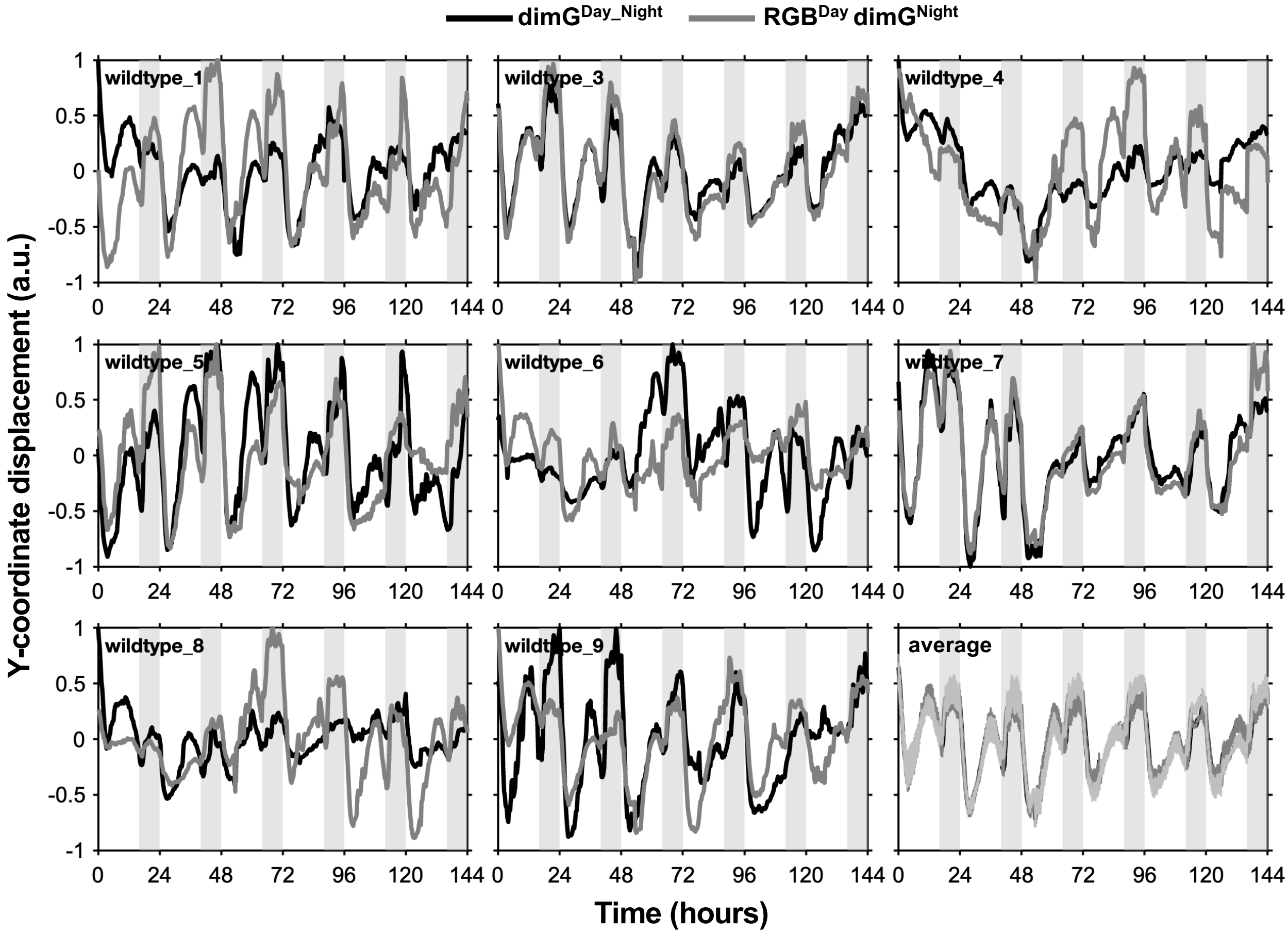

Figure S5 B *ztl* diel oscillations of Y-coordinate displacement for individual plants using dimG^{Day_Night} and RGB^{Day}_dimG^{Night} image sequences under LD

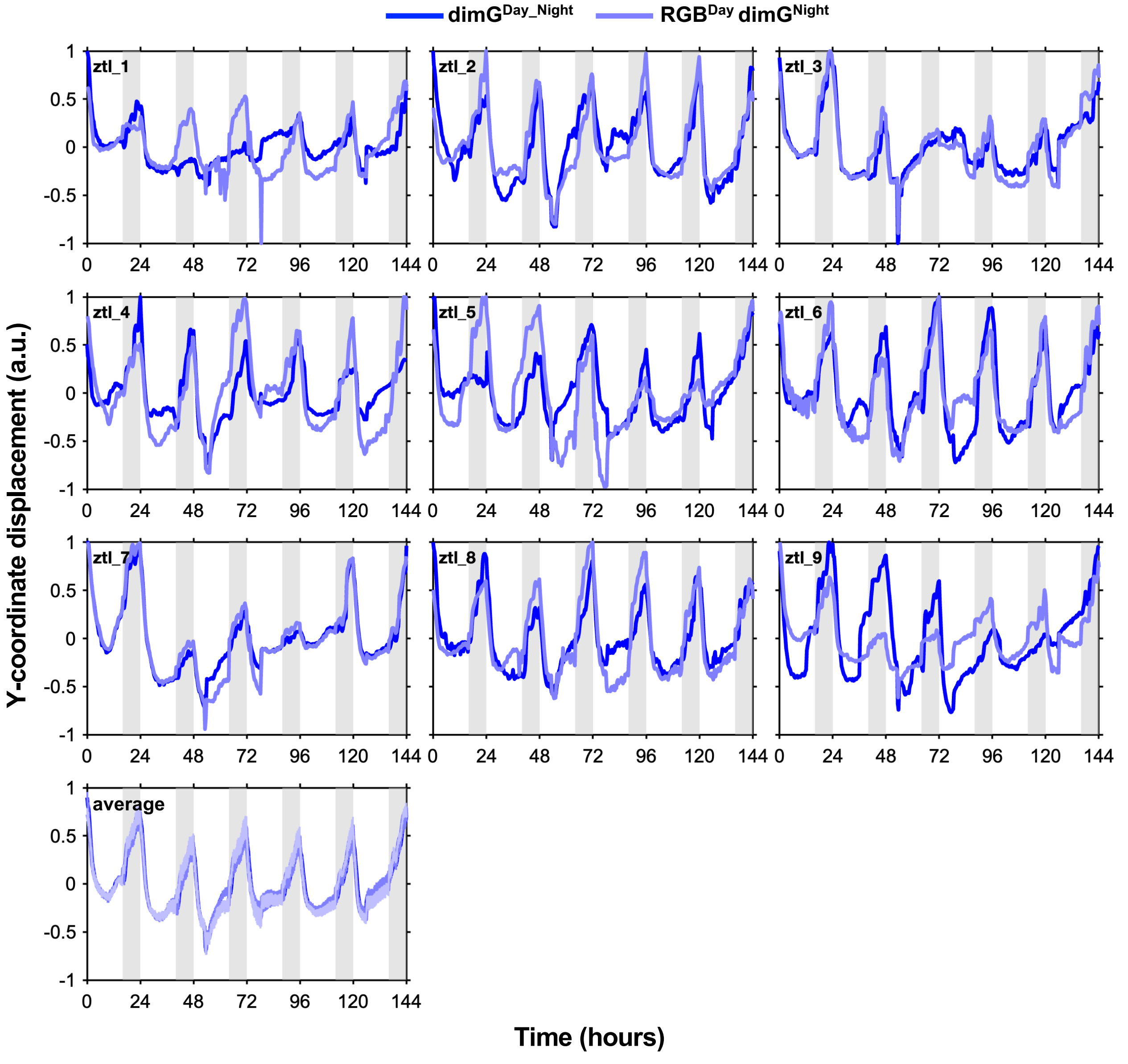

Figure S5 C *lwd1 lwd2* diel oscillations of Y-coordinate displacement for individual plants using dimG^{Day_Night} and RGB^{Day}_dimG^{Night} image sequences under LD

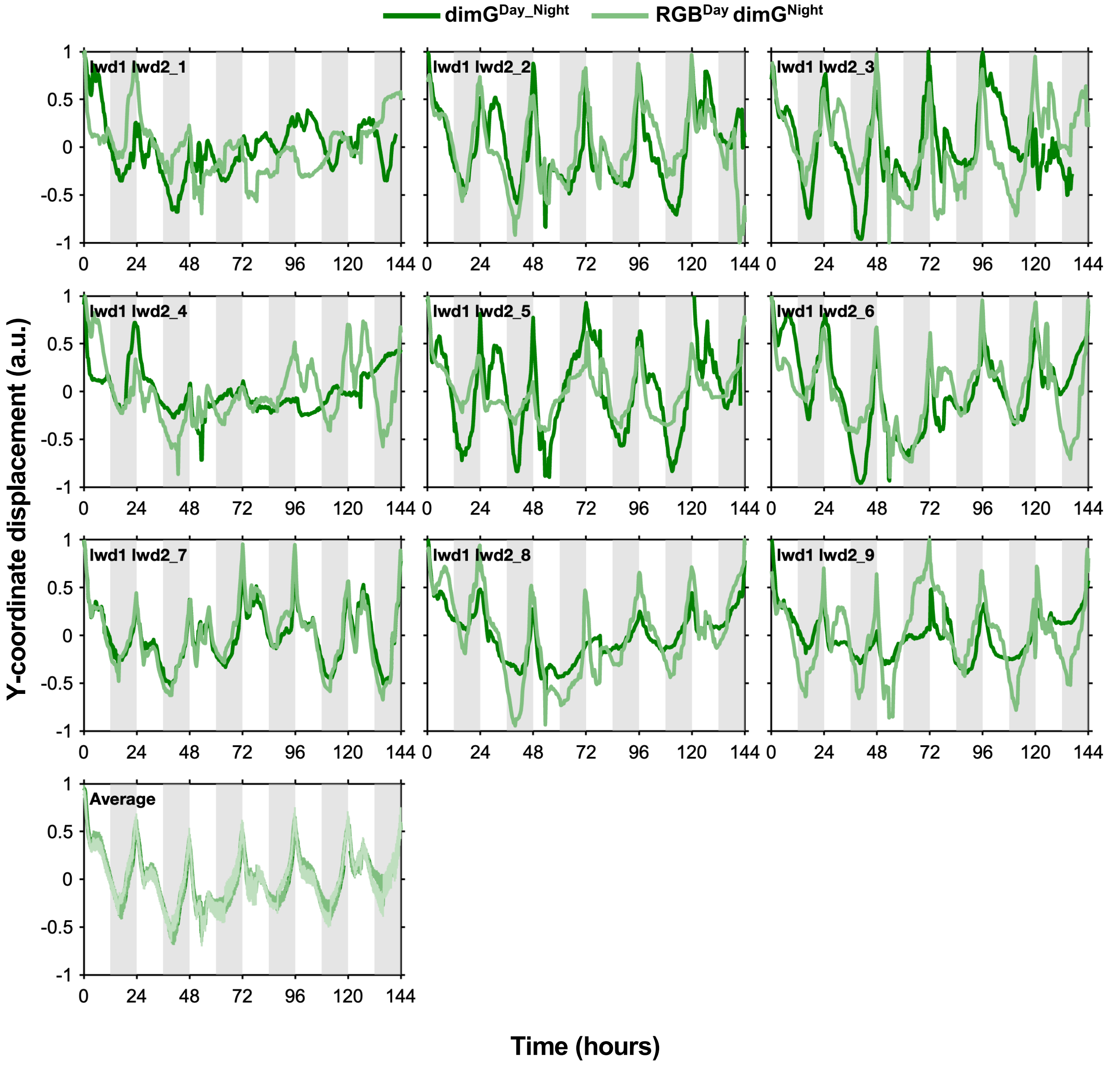

Figure S5 D *elf3* diel oscillations of Y-coordinate displacement for individual plants using dimG^{Day_Night} and RGB^{Day}_dimG^{Night} image sequences under LD

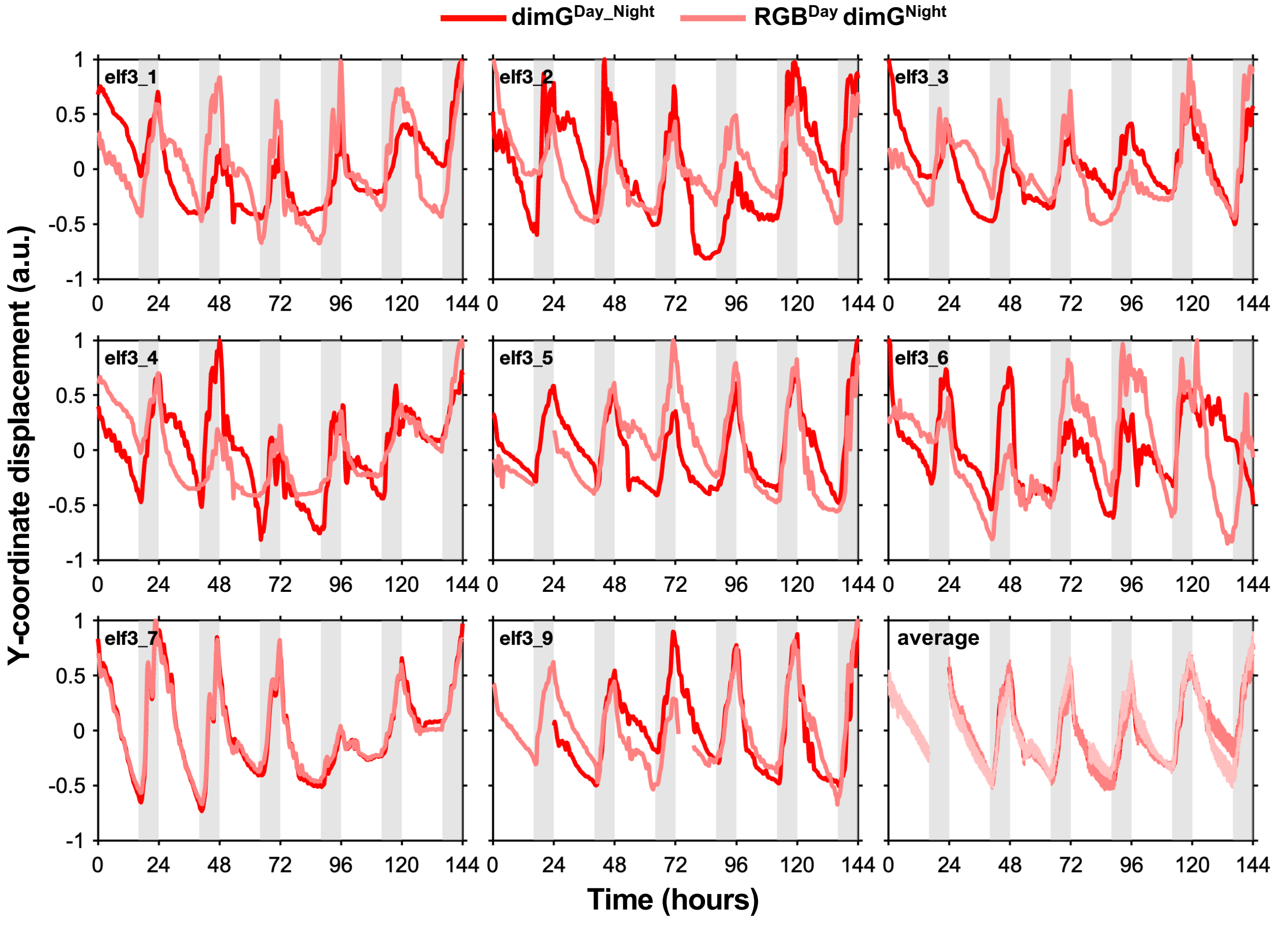

Figure S6. Comparison of IR vs dimG for night imaging, second replicate dataset.

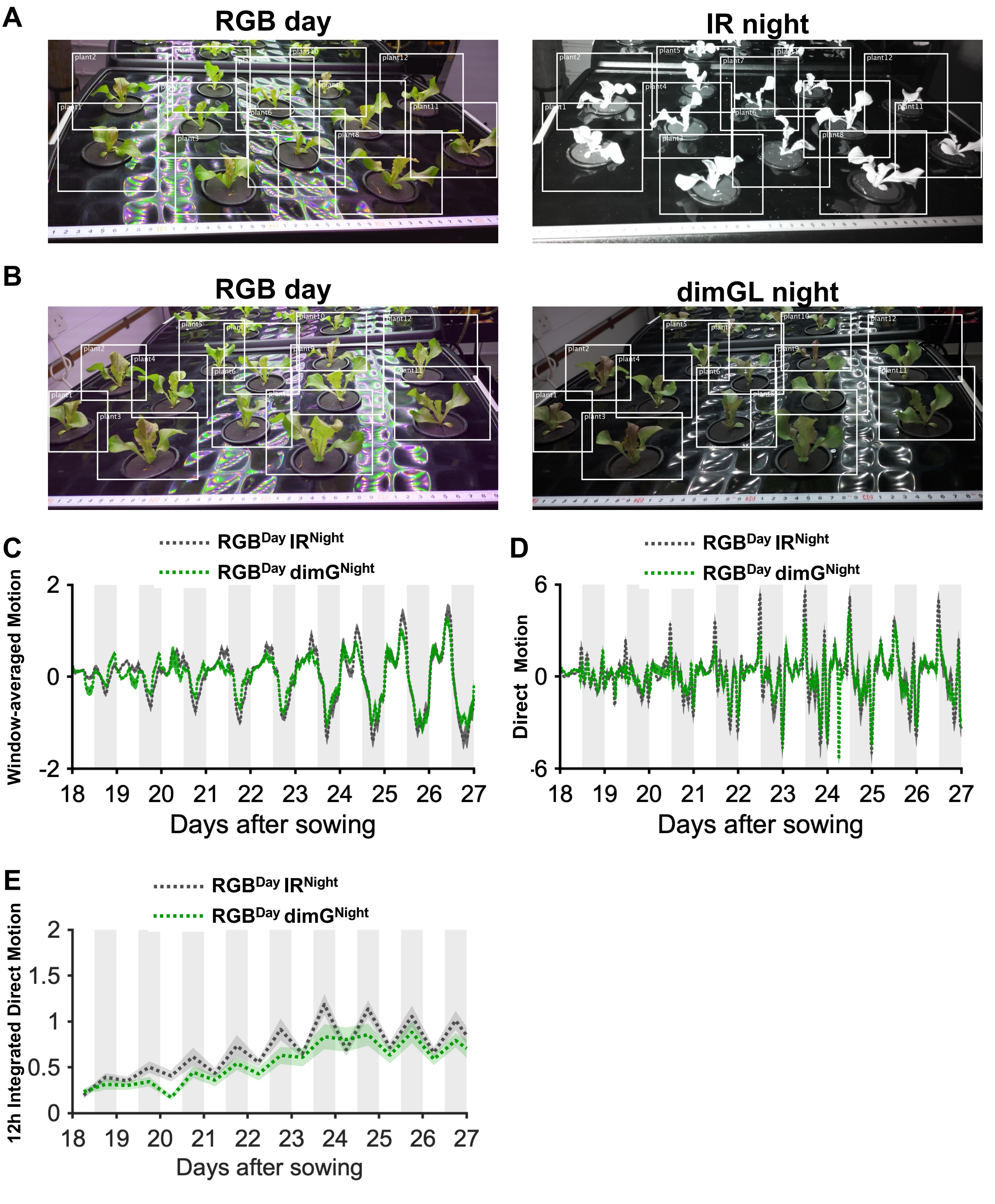

Figure S7 A. Window-averaged motion comparison of IR vs dimG for night imaging (replicate 1)

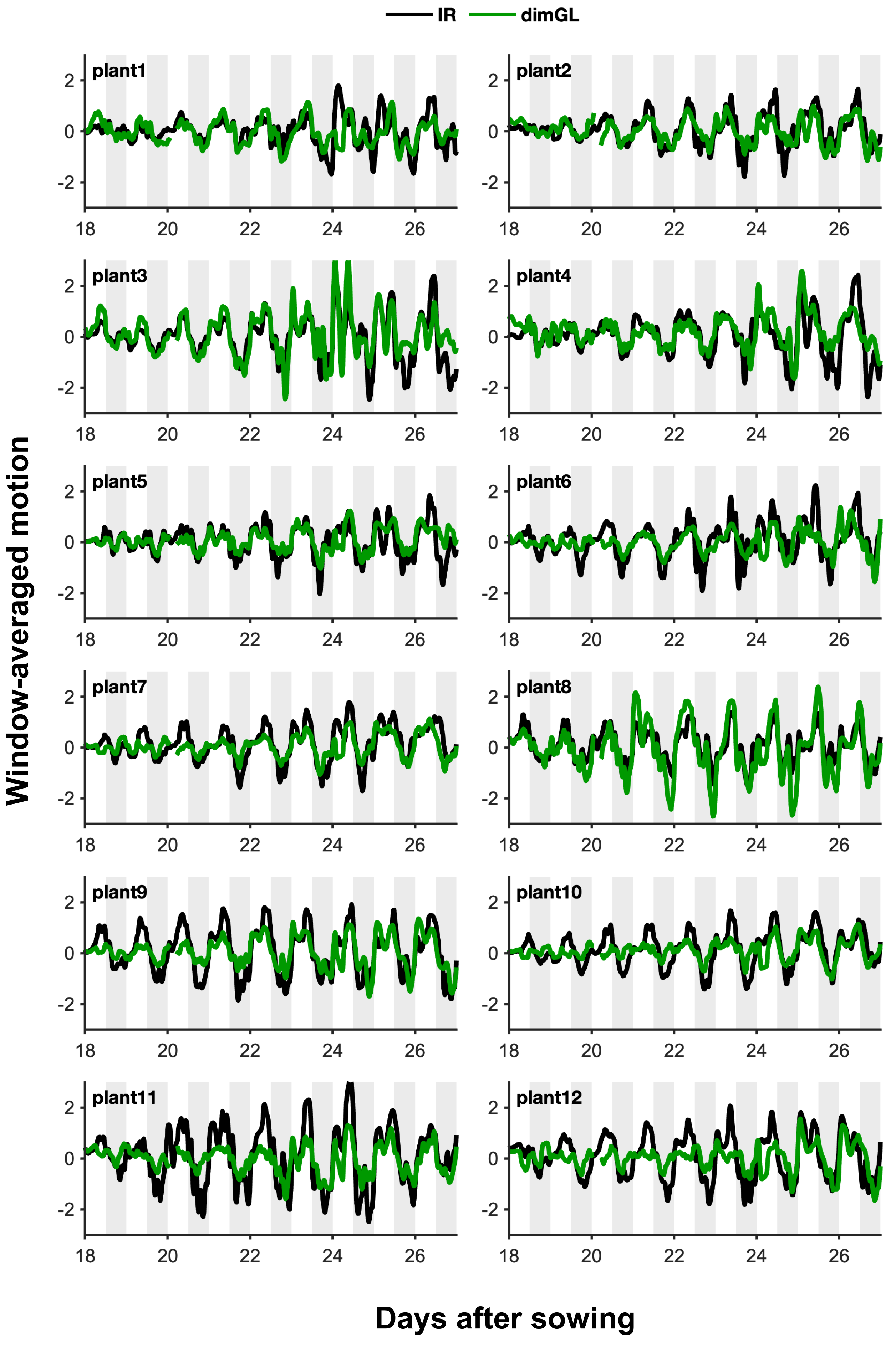

Figure S7 B. Window-averaged motion comparison of IR vs dimG for night imaging (replicate 2)

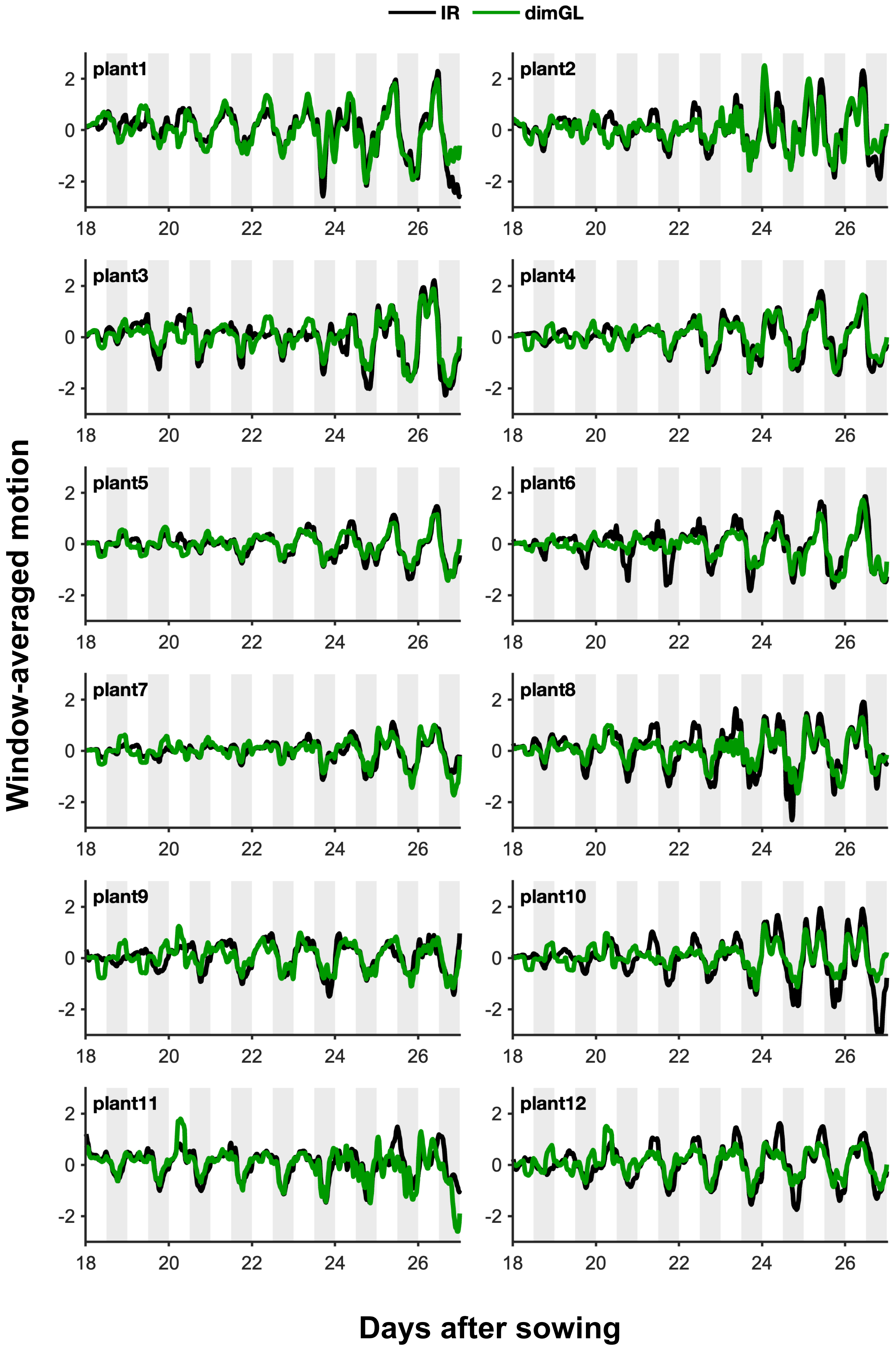

Figure S8 A. Direct motion comparison of IR vs dimG for night imaging (replicate 1)

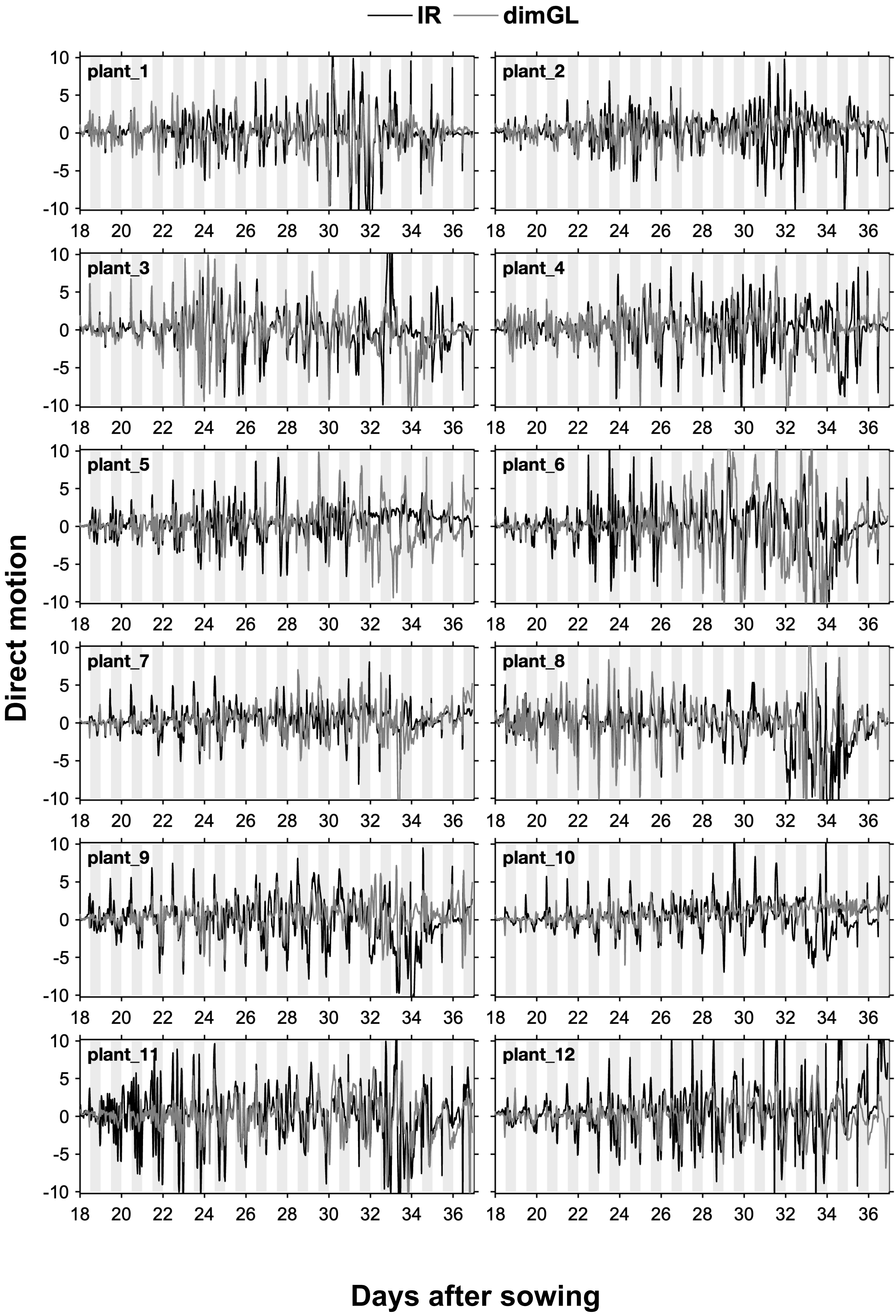

Figure S8 B. Direct motion comparison of IR vs dimG for night imaging (replicate 2)

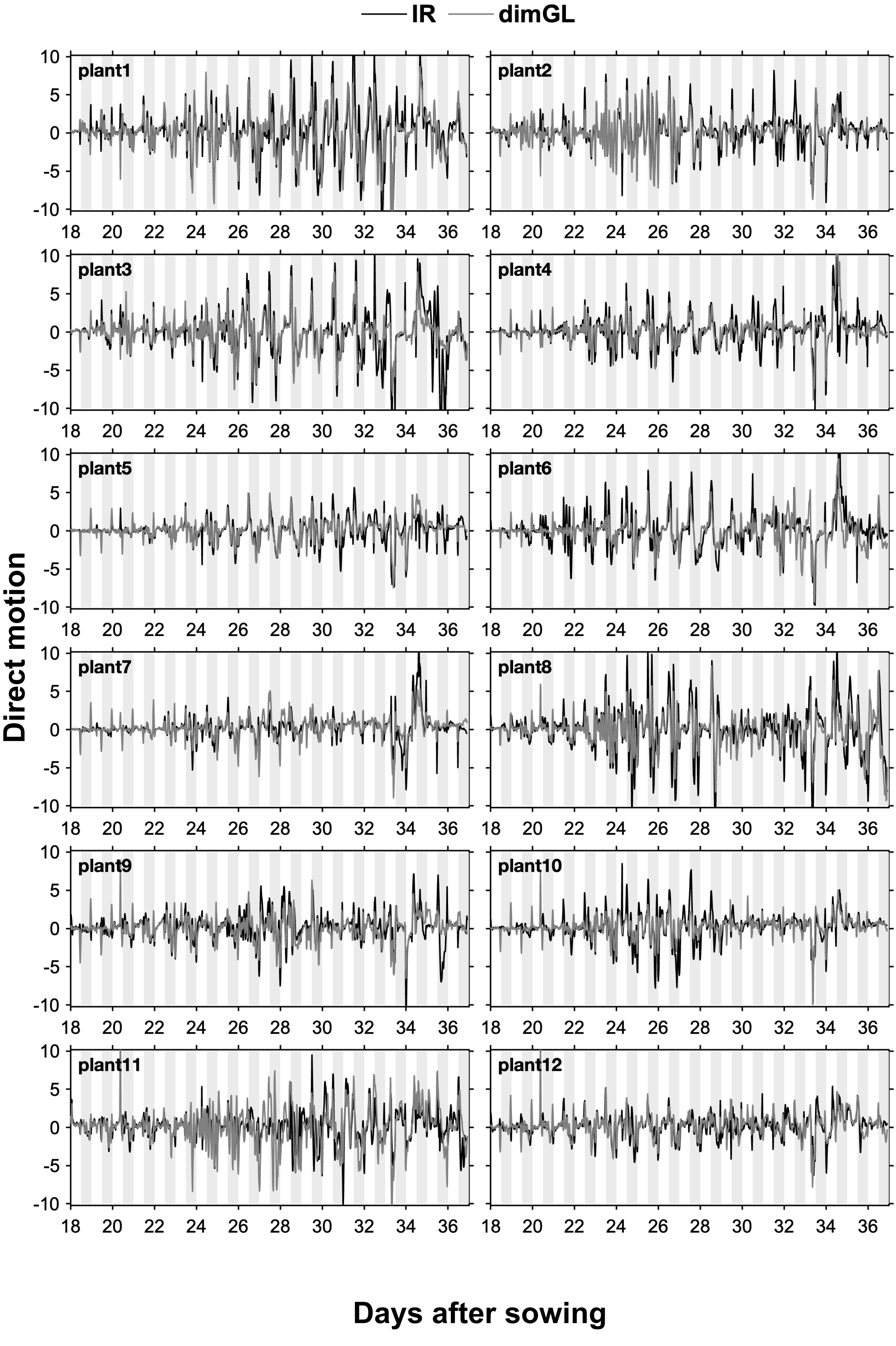

Figure S9. Higher leaf motion at night and increasing trend across the experiment

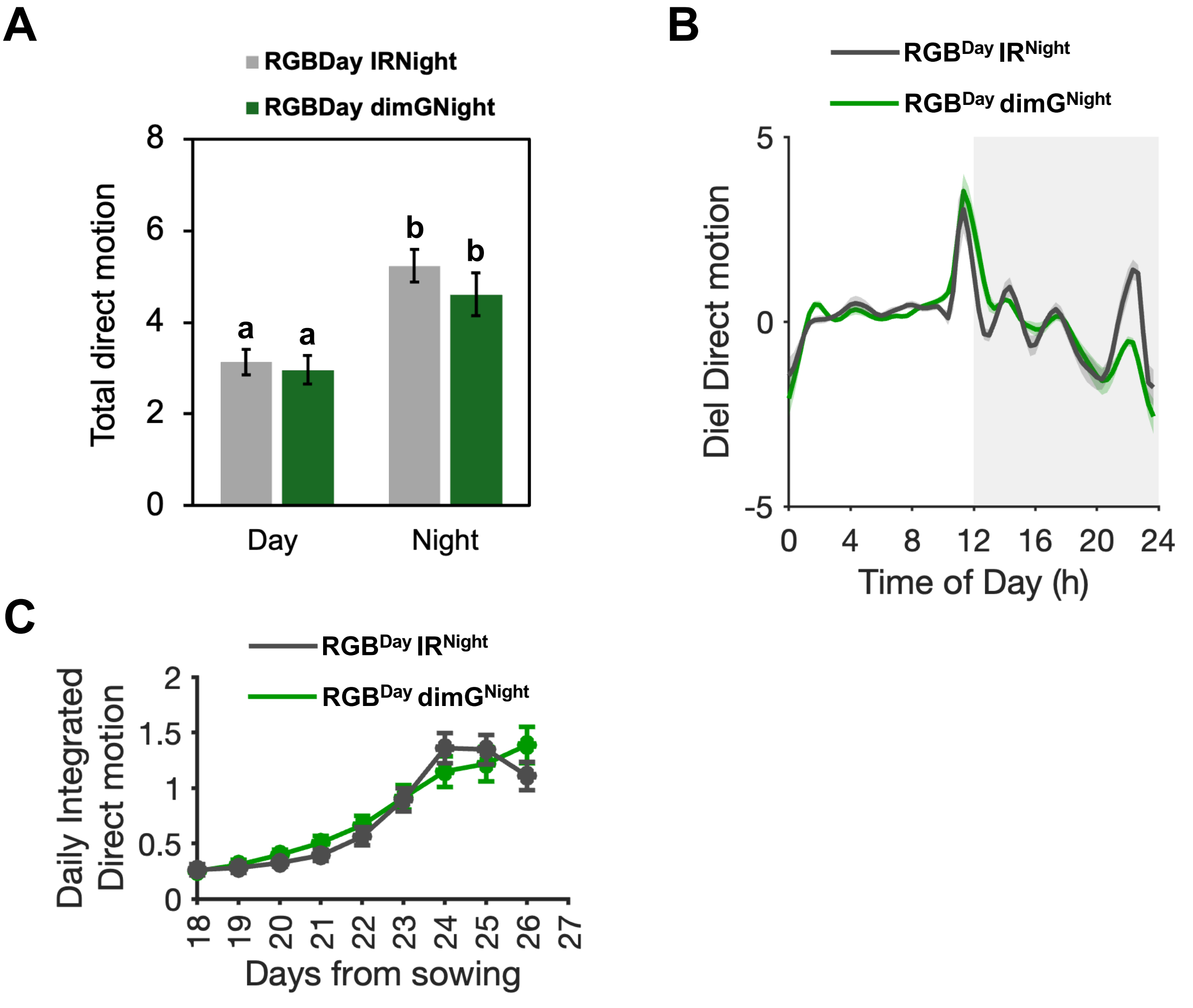

Figure S10. Nighttime exposure to continuous dimG does not influence leaf motion or overall plant growth in lettuce, second replicate dataset.

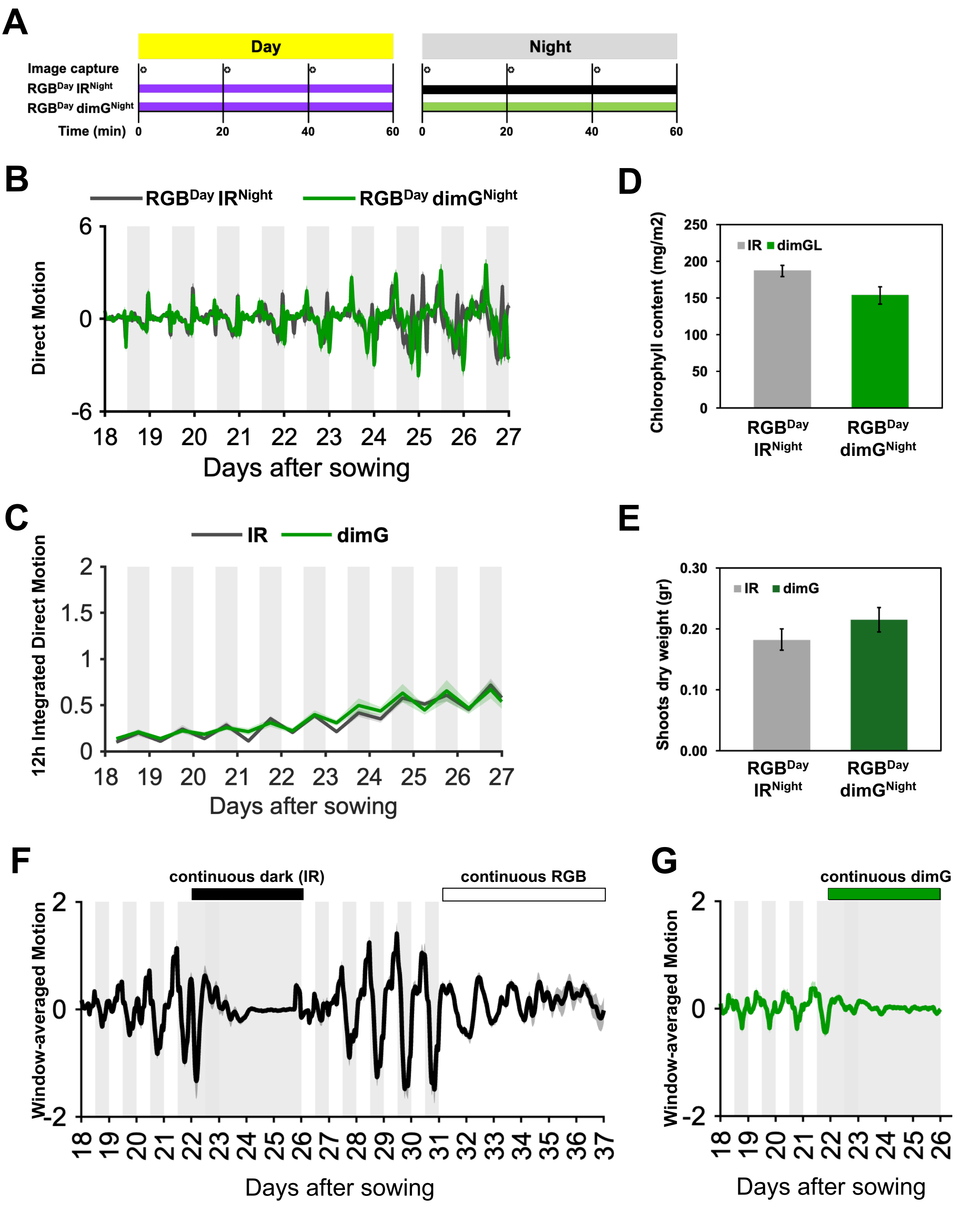

Figure S11 A. Direct motion for individual plants grown under dimG at night. (Replicate 1, Figure 5 B)

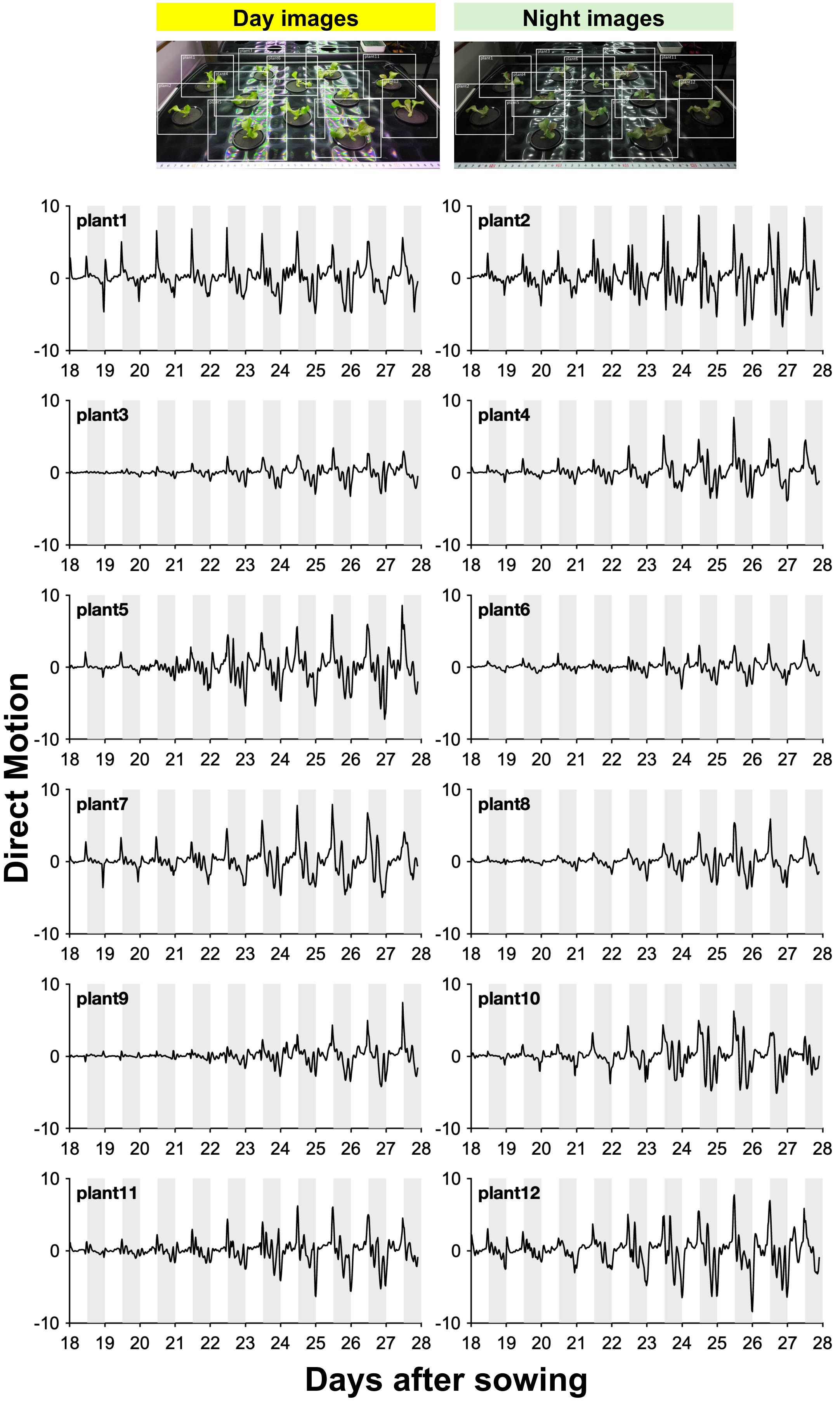

Figure S11 B. Direct motion for individual plants grown under darkness (IR) at night, replicate 1, Figure 5 B)

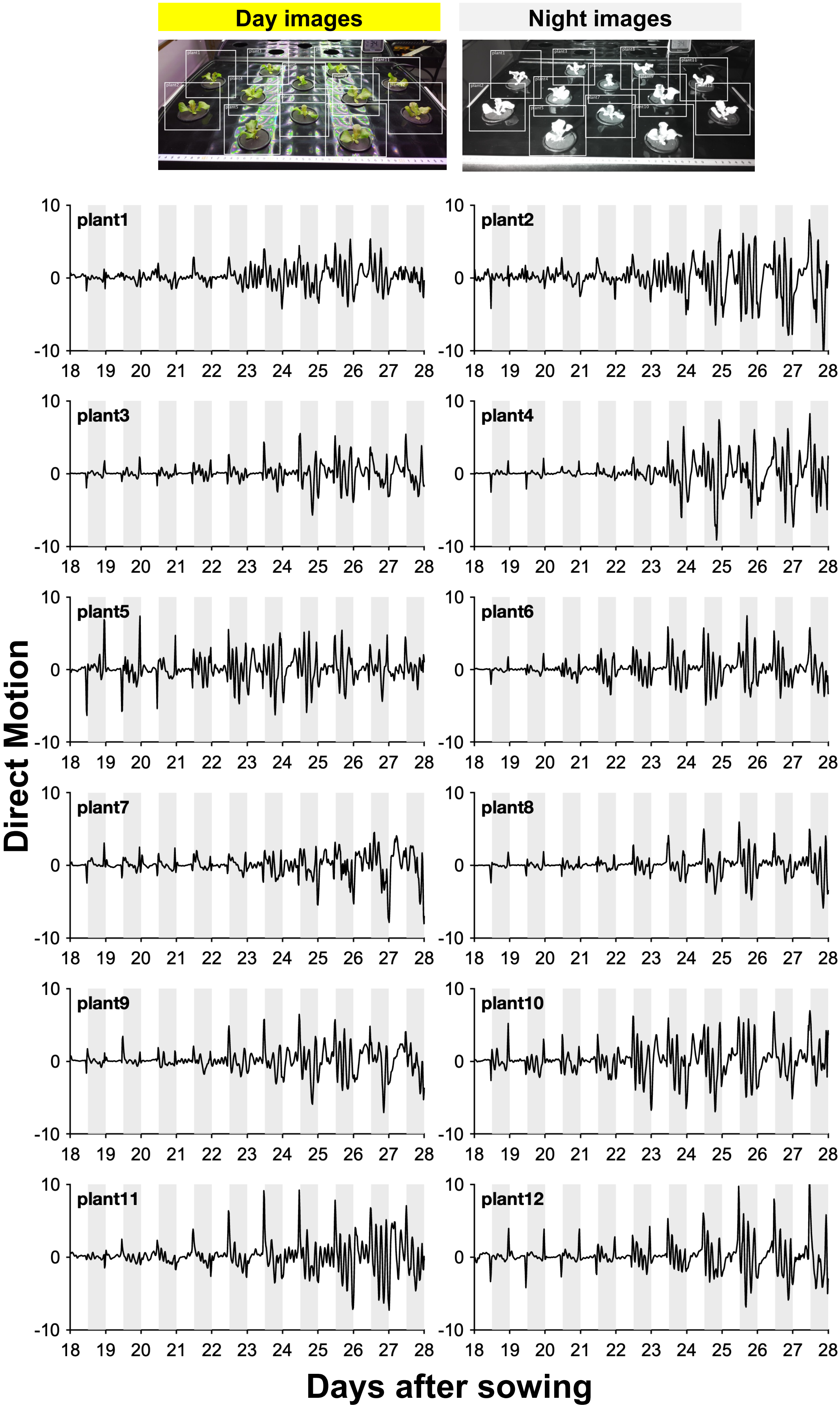

Figure S11 C. Direct motion for individual plants grown under dimG at night. (Replicate 2, Figure S8B)

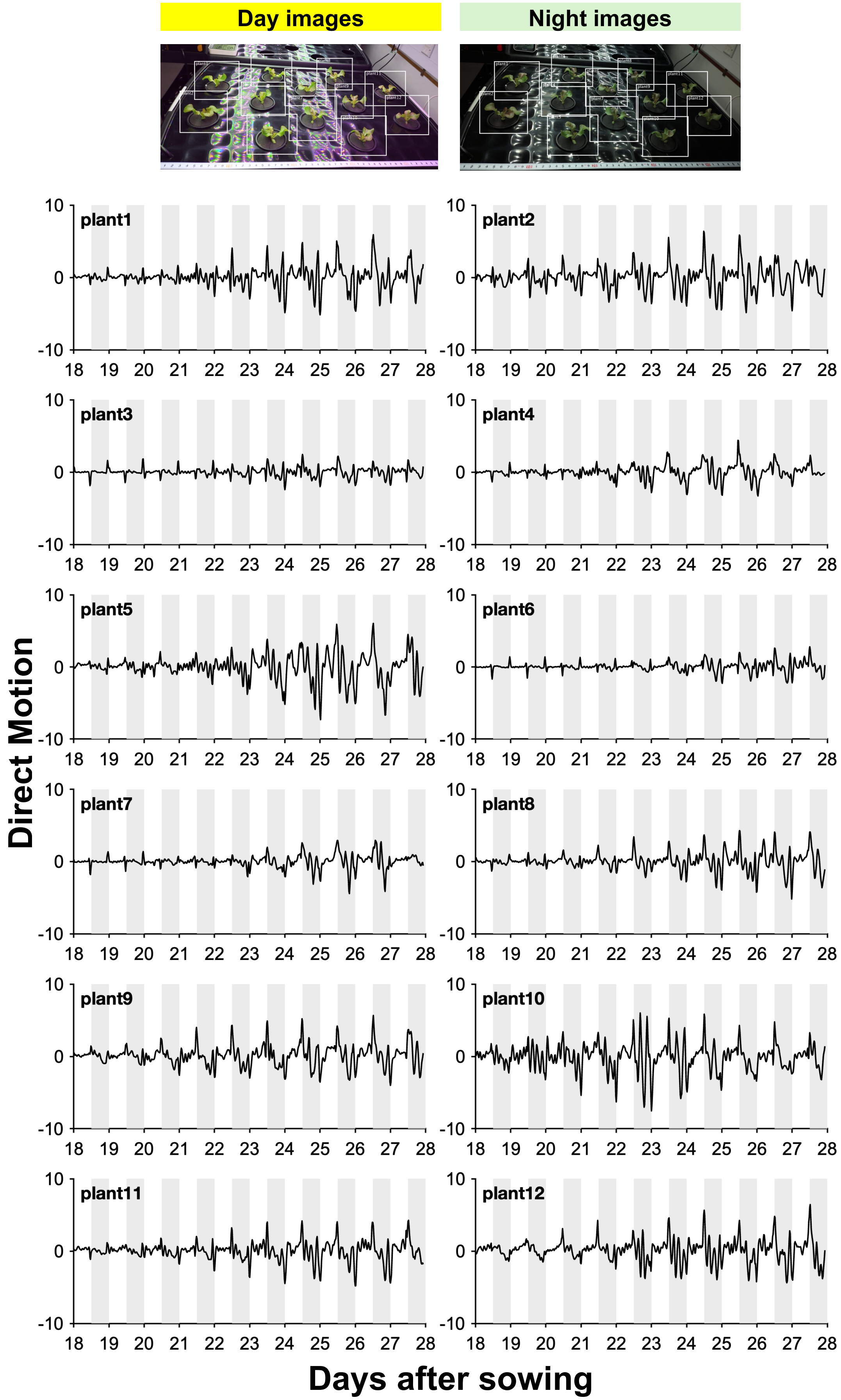

Figure S11 D. Direct motion for individual plants grown under darkness (IR) at night. (Replicate 2, Figure S8B)

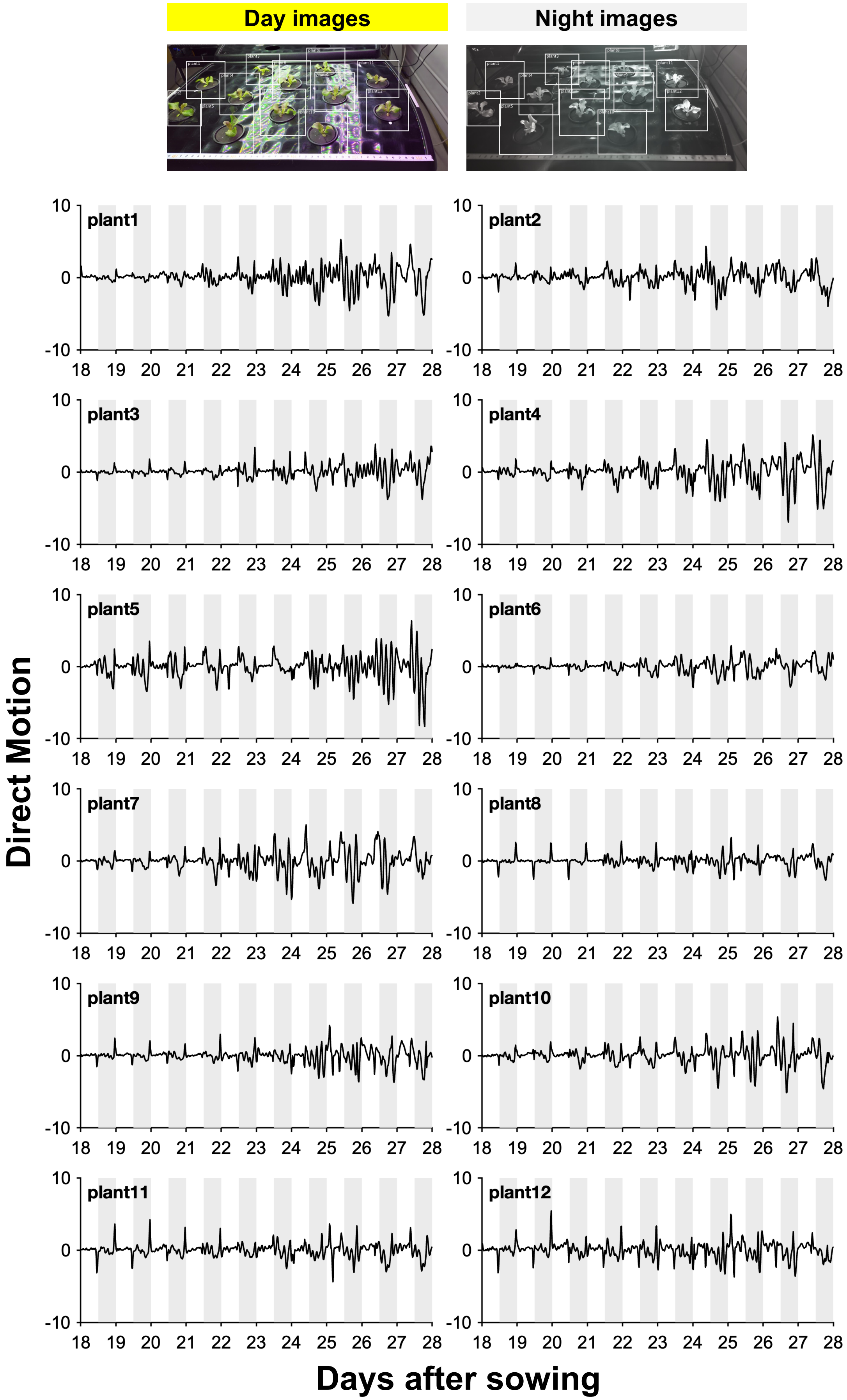

Figure S12 A. DD and LL experiment (Replicate 1, Figure 5 F)

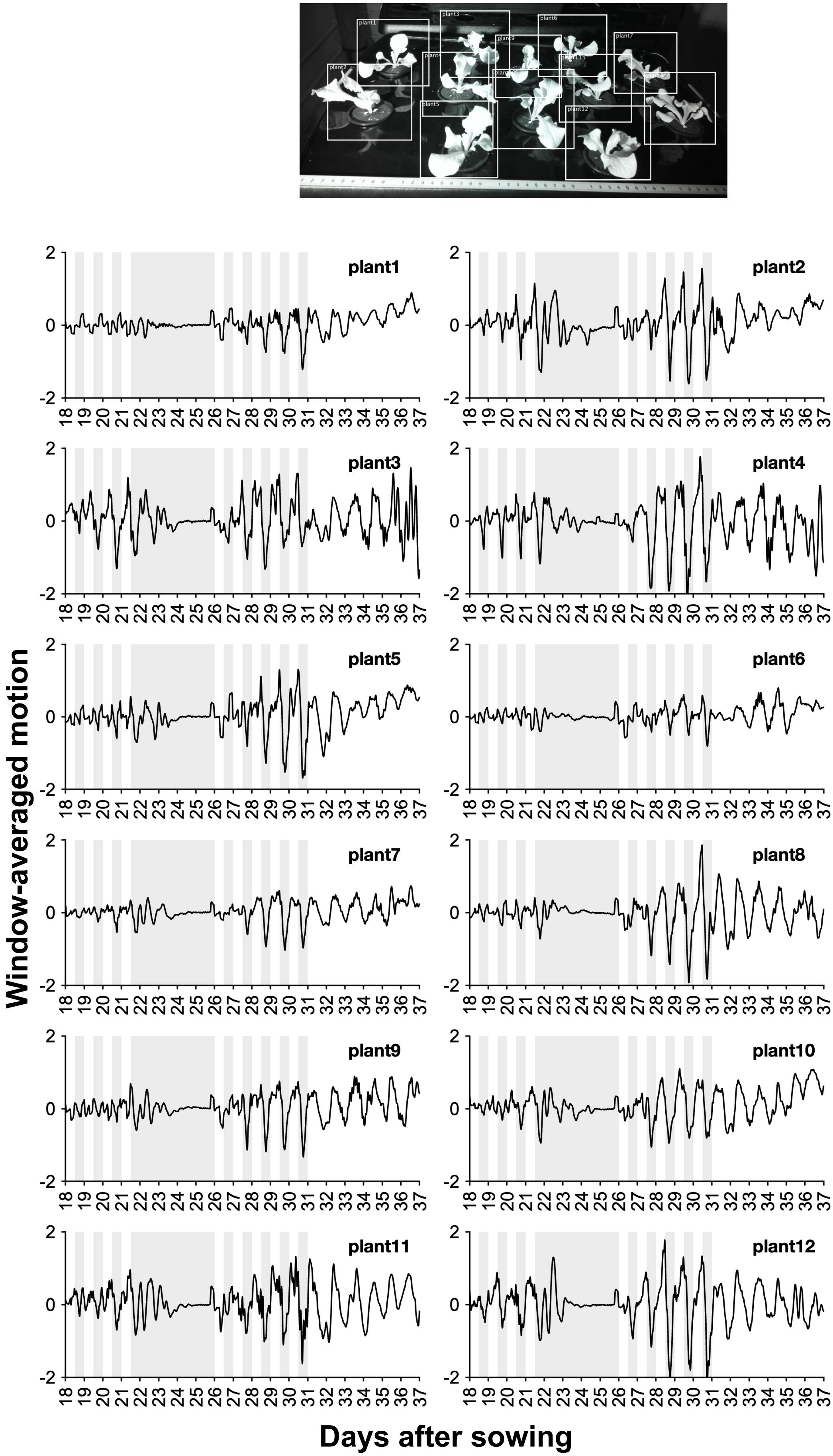

Figure S12 B. DD and LL experiment (Replicate 2, Figure S8 F)

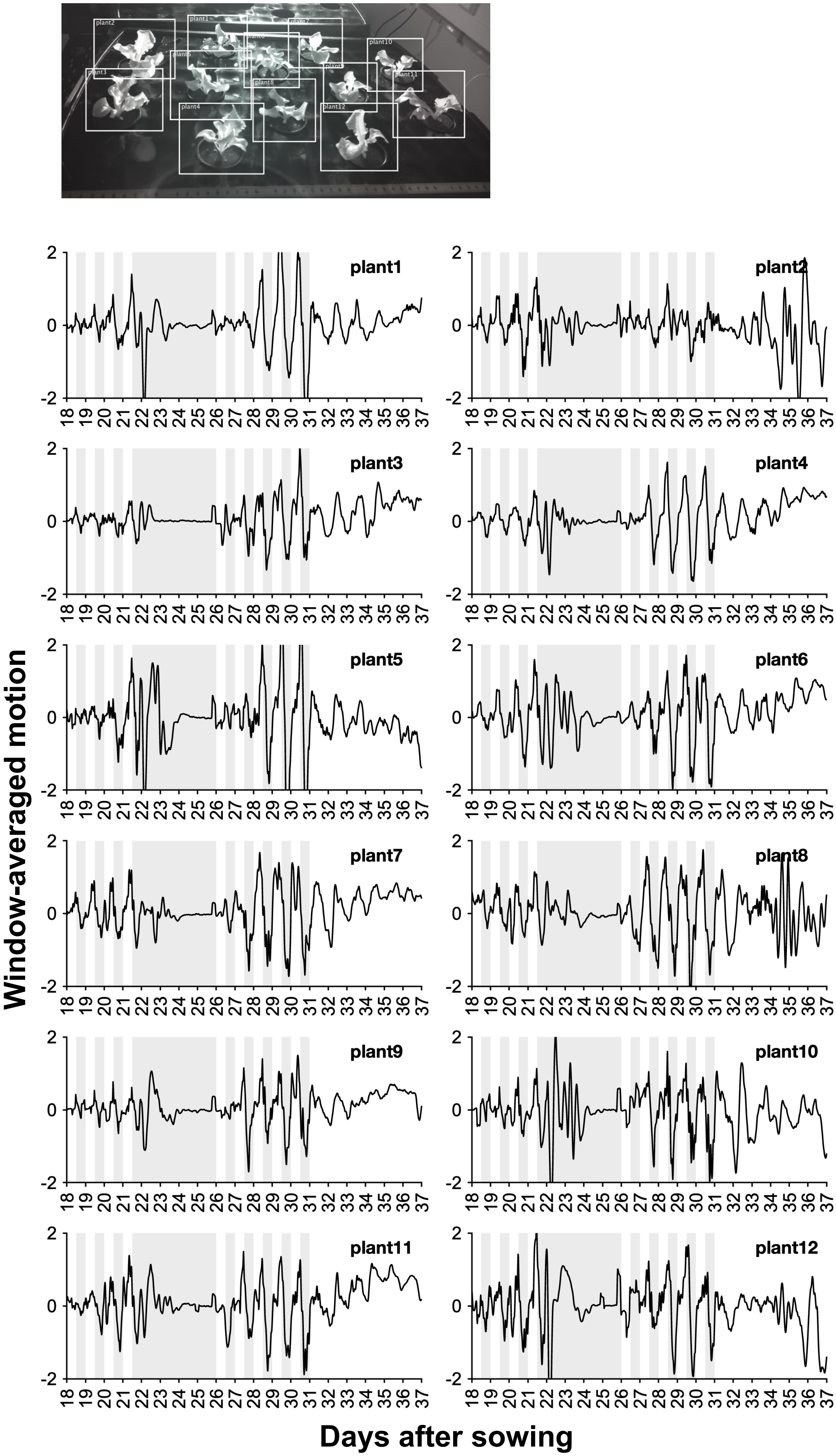

Figure S12 C. continuous dimG (Replicate 1, Figure 5 F; replicate 2 Figure S8F)

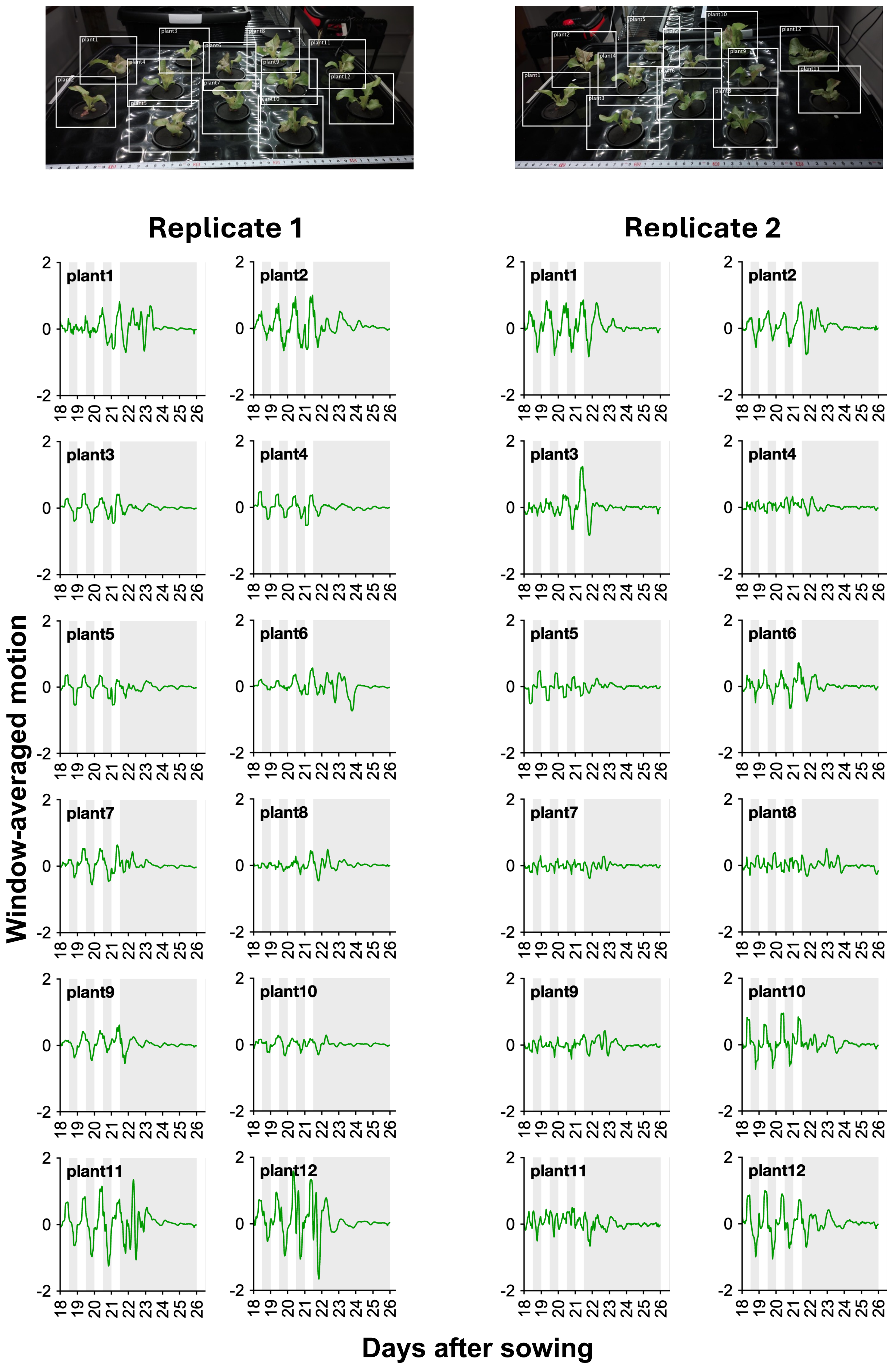

Supplement: Supplementary file 1 [file TPJ-127-0-s001.pdf]
